# tRFs and tRNA Halves: Novel Cellular Defenders in Multiple Biological Processes

**DOI:** 10.3390/cimb44120405

**Published:** 2022-11-28

**Authors:** Jiani Hou, Qianqing Li, Jun Wang, Wenfa Lu

**Affiliations:** 1 Jilin Provincial International Joint Research Center of Animal Breeding & Reproduction Technology, Jilin Agricultural University, Changchun 130118, China; 2 Key Lab of the Animal Production, Product Quality and Security, Ministry of Education, Jilin Agricultural University, Changchun 130118, China; 3 College of Animal Science and Technology, Jilin Agricultural University, Changchun 130118, China

**Keywords:** tRNA-derived small RNA, tRNA-derived fragments, tRNA halves, oxidative stress, apoptosis

## Abstract

tRNA fragments derived from angiogenin or Dicer cleavage are referred to as tRNA-derived fragments (tRFs) and tRNA halves. tRFs and tRNA halves have been identified in both eukaryotes and prokaryotes and are precisely cleaved at specific sites on either precursor or mature tRNA transcripts rather than via random degradation. tRFs and tRNA halves are highly involved in regulating transcription and translation in a canonical or non-canonical manner in response to cellular stress. In this review, we summarize the biogenesis and types of tRFs and tRNA halves, clarify the biological functions and molecular mechanisms of tRNA fragments in both physiological and pathological processes with a particular focus on their cytoprotective roles in defending against oxidation and apoptosis, and highlight their potential application as biomarkers in determining cell fate.

## 1. Introduction

Transfer RNAs (tRNAs) act as cellular deliverers for specific amino acids corresponding to mRNA codons to carry them to the ribosome for protein synthesis [1]. Furthermore, the biological functions of tRNA depend on its various chemical modifications. Over the past 40 years, at least 150 post-transcriptional modifications have been discovered in tRNAs. Unique tRNA modifications allow interactions with endonucleases. These highly modified tRNAs may represent diverse non-canonical functions of tRNAs [2,3]. Moreover, tRNA plays a central role in RNA modification, and the majority of RNA modifications have been detected in tRNAs. tRNA also serves as one of the main sources of small non-coding RNAs with unique and diverse functions.

Cleavage of tRNAs into smaller RNA fragments by endonuclease Dicer or angiogenin (ANG) results in the production of tRNA-derived small RNA fragments (tRFs) and tRNA halves (tiRNAs). The discovery of tRFs and tiRNAs has been traced back to the late 1970s; however, the initial interest in these molecules was limited as they were classified as random tRNA degradation products. Differential expression of tRNA genes regulate the abundance of tRFs and tiRNAs in a non-canonical manner [4,5,6]. Since the discovery of tRFs in viruses [7], archaea (i.e., *Haloferax volcanii*) [8], bacteria [9], plants [10], and mammals [11], they have been found to play roles in cell proliferation, stress, immunity, apoptosis, and other biological processes. A new research hotspot has emerged exploring the biological processes and cellular functions of tRFs and tiRNAs and their underlying molecular mechanisms.

In this review, we present the latest research on the biogenesis, biological functions, and mechanisms of action of tRFs and tiRNAs as regulatory non-coding small RNAs, with a particular focus on their potential regulatory function in apoptosis.

## 2. Classification and Biogenesis of tRFs and tiRNAs

**Figure 1:**
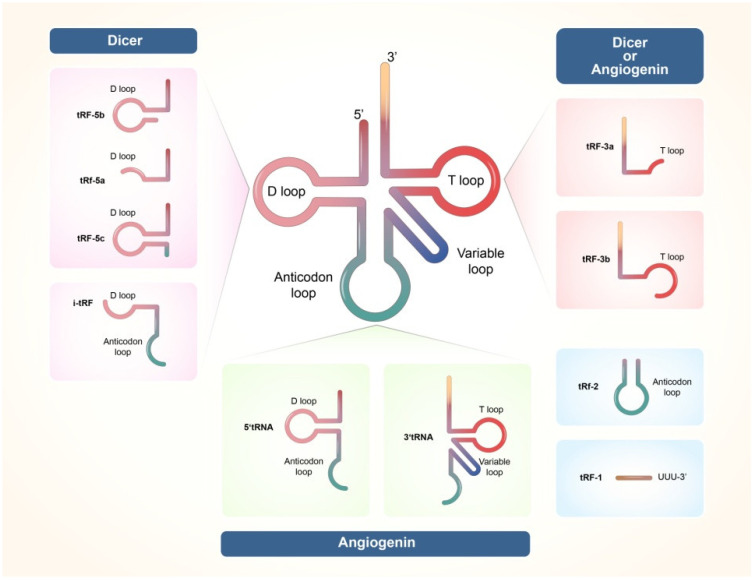
Different types of tRFs and tiRNAs (The figure is cited from Park, J.; Ahn, S.H.; Shin, M.G.; Kim, H.K.; Chang, S. tRNA-Derived Small RNAs: Novel Epigenetic Regulators. [12].

High-throughput sequencing technology exploring non-coding small RNAs has specifically identified two main types of tRNA cleaved fragments based on their different lengths, cleavage sites, and relevant enzymes (Figure 1) [12].

### 2.1. Types of tiRNAs

Certain clusters of cleaved tRNA fragments with a length between 31 and 38 nucleotides (nts) have been observed in human fetal hepatic tissue. Interestingly, these tRNA fragments lack 5′ leader and 3′ trailer sequences found in pre-tRNA, consistent with being ‘halves’ of tRNAs cut at the anticodon site. This suggest that these fragments are specifically cleaved within the anticodon loop of mature tRNAs by a specific endonuclease, and not randomly degraded copies as previously believed [13,14]. These tRNA fragments are defined as tiRNA or tRNA halves with lengths between 28 and 36 nts, representing tRNA-derived or stress-induced small RNAs. Based on the cleavage site of the tRNA, tiRNAs are divided into two subtypes: 5′-tRNA halves beginning at the 5′-end and anticodon loop, and 3′-tRNA halves consisting of the 3′-end and anticodon loop [15,16].

The levels of tiRNAs rise with the overexpression of ANG, whereas the knockdown of ANG causes a decrease in the cellular level of tRNA halves. ANG inhibits 5′-tiRNA-induced optimal stress granule (SG) assembly and transfection with the ANG inhibitor RNH1 abrogates this effect in human U2OS cells, suggesting that ANG is responsible for the production of these fragments [13]. The cleavage function of ANG is determined by its subcellular localization. ANG is localized in the nucleolus when the environment is suitable for cell growth; however, when the organism faces stress, ANG rapidly accumulates in the cytoplasm, activates enzymatic activity, and degrades tRNAs into tiRNAs [17]. Furthermore, mutation of ANG at P112L in patients with amyotrophic lateral sclerosis, leads to the loss of its nuclear translocation property, preventing tiRNA production [18]. The addition of ANG in vitro also mediates tiRNA production, which is consistent with in vivo findings [19]. Lastly, specific tRNA modifications impact endonuclease activity. Rny1p, a member of the RNase T2 family, rescues yeast cell survival from oxidative stress by being released in the cytosol [20].

tiRNAs are believed to act as stress fragments as they are cleaved in response to stresses, including phosphate deficiency, amino acid starvation, heat shock stress, environmental hypoxia, and oxidative stress, ultimately resulting in cell death [21,22,23]. However, other reports indicate that tiRNAs are not detected in response to the cleavage of tRNAs induced by all stress conditions. 

### 2.2. Types of tRFs

Cleaved tRNA fragments that map to known tRNA genes are called tRFs. tRFs are generated at the 5′- or 3′-end of mature tRNAs or trailer sequences of precursor tRNAs with lengths between 14 and 30 nts [24]. 

tRFs mainly consist of four types: tRF-5, tRF-3, tRF-2, and tRF-1 according to their mapped positions on tRNA, while other tRFs are referred to as internal tRFs (i-tRF) [25,26,27]. tRF-5s are cleaved in the D loop or D stem by Dicer, or occasionally at the 5′ half of the anticodon stem.

Fifteen, twenty-two, and thirty-two nts are the three most abundant lengths of tRF-5s when separately counting unique plotted tRF reads of different lengths in tRFs and tiRNAs sequencing. Therefore, tRF-5s are divided into three specific subtypes: (1) tRF-5a (14–16 nts), (2) tRF-5b (22–24 nts), and (3) tRF-5c (28–30 nts). tRF-3 is cleaved by Dicer, ANG, or other ribonucleases in the TΨC loop, and is commonly 13 to 22 nts in length followed by a CCA tail sequence with two peaks at tRF-3a (18 nts) and tRF-3b (22 nts). The precursor tRNA is degraded at the 3′-end by RNase Z or its cytoplasmic homologue, ELAC2, to generate tRF-1. tRF-1 has a broad length distribution ranging from 12 to 36 nts because tRF-1 usually ends with a RNA polymerase III transcription termination signal and contains different tails in each pre-tRNA, including UUUUU, UUCUU, GUCUU, and AUCUU [28,29]. Additionally, there is a distinct type of tRF identified in breast cancer that only consists of anticodon stem–loop nucleotide sequences when exposed to hypoxic conditions [30]. This mature tRNA fragment is classified as tRF-2. tRFs that do not belong to the traditional classifications and regulations are also referred to as i-tRFs [31].

Although tRFs are commonly found in eukaryotes and prokaryotes [32,33], not all tRNAs produce tRFs and their reads are not equally abundant. tRF-5 generates more copies than tRF-3, while tRF-1 presents the lowest reads. This difference in abundance may be explained by the presence of a 3′ trailer in the 14–36 nts sequence in the pre-tRNA. For example, *Caenorhabditis elegans* and *Saccharomyces cerevisiae* have accumulated fewer tRF-1 copies than humans and mice because these species possess ten-fold fewer pre-tRNA trailer sequences within the 14–36 nts sequence. However, tRF-1 may be negatively correlated with the number of 3′ trailers in *Drosophila* and *Schizosaccharomyces pombe*, suggesting that additional factors affect tRF-1 abundance. Conversely, tRF abundance is tissue-specific. tRF-5 and -3 are present at similar abundances in ovaries and embryos, such that their expression is higher in embryos than in the testes and brain in mice. In contrast, the number of tRF-1 cloning copies is higher in the brain than in embryos and embryonic stem cells [34].

## 3. Mechanisms of Action of tRNA-Derived Small RNAs (tsRNAs)

The generation of tsRNAs is significantly site-specific and restricts certain isoforms of tRNA [12]. Importantly, tsRNAs are characterized by functional regulation of non-coding small RNAs.

### 3.1. Regulating mRNA Stability

Photoactivatable ribonucleoside-enhanced crosslinking and immunoprecipitation (PAR-CLIP) and crosslinking, ligation, and sequencing of hybrid data suggest that some tRFs represent a seed region and directly bind to mRNA to regulate mRNA stability. For example, tRF-1s derived from tRNA-Gly-CCC directly silenced the Timp3 gene by interacting with its 3′-UTR in a model of myocardial hypertrophy in rats [35]. Moreover, luciferase activity in the wild-type FBXO47 3′-UTR significantly decreased following the transfection of tRF-3019a mimics in gastric cancer [36]. tiRNAs showed the similar function way, for example, 5′-tiRNA-Val, may reduce FZD3 expression by targeting its 3′-UTR in breast cancer cells [37]. PAR-CLIP data also indicate that tRF-3 and -5 interact with AGO proteins, specifically AGO-1, -3, and -4 to post-transcriptionally impact gene expression. A tRF-3009a fragment originating from tRNA-Leu-TAA is loaded into Argonaute and interacts with GW182/TNRC6A to mediate mRNA degradation in a Dicer-independent manner [38]. Furthermore, tRF5-Glu-CTC impairs the gene trans-silencing function when cells are deficient in AGO-1 or -4 in respiratory syncytial virus (RSV) replication and host gene regulation [39]. Some tRF-3s downregulate target genes via binding to their 3′-UTRs and subsequently recruiting AGO proteins to form the RNA-induced silencing complex (RISC). For example, tRF-3003a derived from tRNA-Cys-GCA targets the JAK3 3′-UTR and is enriched in AGO/RISC formation in chondrocytes [40]. The tRF-3017A similarly regulates NELL2 in gastric cancer [41]. Despite the canonical or non-canonical miRNA mechanisms described above, some tRFs bind with RNA-binding proteins to alter mRNA stability. tRF-2s, derived from tRNA-Glu, tRNA-Asp, tRNA-Gly, and tRNA-Tyr, displace the 3′-UTRs of oncogenic transcripts from the RNA-binding protein YBX1 by co-complementary sequence, and thus suppress their mRNA stability in breast cancer cells [30]. tRF3E specifically interacts with nucleolin and represses the translation of p53 mRNA in breast cancer [42]. Here, we see that tRFs utilize various approaches to regulate mRNA stability; therefore, constructing an inner regulation network may increase our understanding of tRFs and their mRNA targets.

### 3.2. Regulating Translation

Lives are exposed to environmental stress, and environmental stress exerts significant effects on biological organization. Therefore, the defense against cellular damage caused by extreme climate, ultraviolet irradiation, and environmental stress is crucial for organisms to survive. The reprogramming of protein translation occurs in response to cellular stress to adjust adverse environmental conditions, and this repair process targets translational machinery. Stress-induced translational inhibition via blockage of the assembly of the 48S initiation complex induces eukaryotic initiation factor-2α (eIF2α) phosphorylation and SG assembly to inhibit global protein synthesis and subsequently trigger mRNAs that encode “housekeeping” proteins [43,44]. Stress-induced tRNA fragments mediate translational repression. Phloem sap-specific tRNA fragments inhibit translation in an non-specific manner and likely interfere with ribosomal activity in pumpkins [45]. ANG induces tiRNA as a stress-activated ribonuclease, and the tiRNAs repress protein synthesis without exogenous stress by promoting a phospho-eIF2α-independent stress response in human U2OS cells. 35S-labeled protein autoradiographic analysis further revealed that 5′-tiRNAs, rather than 3′-tiRNAs, inhibit global protein synthesis [13]. Similarly, Emara et al. [19] concluded that 5′-tiRNA-Ala enhances arsenite-induced SG assembly because of the presence of 5′-monophosphates and this translation initiation inhibition is eIF2α phosphorylation-independent. Subsequent studies revealed that tiRNAs assemble into a G-quadruplex-like structure that displaces eIF4G/eIF4A from uncapped RNAs. tiRNAs also substitute eIF4F from isolated m7G caps, while eukaryotic translation initiation factor 4E (IF4E) and IF4E-binding protein 1 are not involved in this process [46,47]. Furthermore, translational silencer YB-1 binds to 5′-tiRNA via the cold-shock domain. Although this interaction is dispensable for tiRNA-mediated translational repression, it is important for assembling tiRNA-suppressed mRNAs into SGs [48]. These findings contribute to our understanding of how tiRNAs, particularly 5′-tiRNAs, target the translation initiation machinery. Unlike tiRNAs, tRFs primarily participate in the elongation phase of translation via different modes. Pseudouridylation (Ψ), the most abundant and widely distributed type of RNA epigenetic regulation in molecules in living organisms, is catalyzed by pseudouridine synthase (PUS) 1, PUS4, and PUS7. In embryonic stem cells, protein biosynthesis increases when PUS7 inactivates and ultimately impairs 5-tRF-mediated translation regulation [49]. In mammals, 5′-tRFs may influence transcript stability as a molecular brake even if complementary target sites in the targeting mRNA are absent [50,51]. In contrast, in Arabidopsis, tRNA fragments function as potential translation modulators [52]. tRFs compete with mRNAs and bind to the small ribosomal subunit of the initiation complex, resulting in global translation attenuation both in vivo and in vitro in the halophilic archaeon *Haloferax volcanii* [53]. These findings suggest that the tRF mechanism for regulating translation is functionally conserved.

## 4. Biological Functions of tsRNAs

### 4.1. Regulating Ribosome Biogenesis

Ribosomes, composed of ribosomal RNA and proteins, are critical for mature ribosomal subunit generation and protein synthesis. tsRNAs are recognized as novel regulators of ribosome biogenesis. In the unicellular eukaryote Tetrahymena, ribosomal RNA processing requires activation of the exonuclease Xrn2 by Twi12, a Tetrahymena thermophila Ago/Piwi protein, and formation of the TXT (Twi12/Xrn2/Tan1) complex with Tan1. The TXT complex is localized in the nucleus and binding between Twi12 and tRF-3s is essential [54]. A tRF-3 from tRNA-Leu-CAG induces apoptosis via binding to ribosomal protein S28 (RPS28) and RPS15, and tRF-3 targets the coding sequence (CDS) and 3′-UTR of RPS28 mRNA, enhances RPS28 expression, and increases 40S ribosomal subunits in hepatocellular carcinoma in mice. As the CDS target site is present in several vertebrates, this may be a conserved regulation site for ribosome biogenesis between species [55,56]. Moreover, a 5′-tRNA-Pro half mediates ribosome stalling and peptidyl-tRNA accumulation and inhibits global protein translation in HeLa cells [57]. These findings contribute to our understanding of tsRNAs as heterogonous ribosome regulators.

### 4.2. Regulating Viral RNA Reverse Transcription

Viral infection may induce tRF production in host cells. tRF5-Glu-CTC represses target mRNA expression in a non-canonical miRNA manner and promotes RSV infection [7]. Furthermore, tRF5-Glu-CTC targets the 3′-UTR of the anti-RSV protein E receptor-2 (APOER2) and interacts with AGO-1, AGO-4, and P protein to control RSV replication [39,58]. Besides tRF5-Glu-CTC, two other tRFS, tRF5-Gly-CCC and tRF5-Lys-CTT, promote RSV replication and affect inflammation during RSV infection [59]. Moreover, a primer-binding site (PBS)-tRNA-Lys3 duplex is formed when human immunodeficiency virus-1 (HIV-1) infects MT4 T-cells. From this duplex, an 18 nt tRF3 acts with protein Argonaute-2 to engage RISC and ultimately downregulate HIV-1 replication [60]. Similarly, tRF-3019 complements the PBS of human T-cell leukemia virus type 1 (HTLV-1) and restricts HTLV-1 reverse transcription [61]. The pre-tRNA 3′ trailer-derived tRF-U3-1 inhibits RNA chaperone La/SSB binding to hepatitis C virus and thus mediates viral gene expression [7]. Based on these findings, clinical applications of tRFs as diagnostic markers and therapeutic targets are expected.

### 4.3. Regulating Epigenetic, Genetic, and Intergenerational Inheritance

As transposable elements (TEs) contribute to genome instability, mammalians have evolved unique epigenetic mechanisms to prevent the impact of TEs [62]. Importantly, tRFs exhibit high abundance in mouse stem cells. Small RNA sequencing analysis has revealed that a 22 nt tRF containing a 3′-CCA tail targets the tRNA PBS and functions in endogenous retrovirus (ERV) reverse transcription. A different 18 nt tRF specifically interferes with long terminal repeat (LTR) retrotransposons. LTR retrotransposons and ERVs are mainly responsible for novel insertions in the genome, and tRFs may potentially conserve small RNAs that maintain epigenetic stability [63]. In addition to the tRF targeting mechanism, Piwi-interacting RNAs (piRNAs) mediate transposon silencing during epigenetic reprogramming. The action of tsRNAs is similar to that of piRNAs, and therefore, tRFs are likely novel epigenetic regulators in determining cell fate. Dysregulation of tsRNAs is strongly involved in human cancers, including two tRFs, ts-3676 and ts-4521, that interact with Piwi proteins in chronic lymphocytic leukemia [64,65]. Couvillion et al. [66] demonstrated that Twi12 selectively binds to a tRF-3 fragment with a 3′-CCA tail (1822 nts in length) which is essential for cell growth and improving translational fidelity in Tetrahymena. 

Recent research on the epigenetic regulatory function of tRFs has been focused on metabolic diseases. For example, tRF-Glu-TTC suppresses adipogenesis by promoting preadipocyte proliferation [67]. Interestingly, sperm that generate tRNA fragments, including both tRFs and tiRNA, show epigenetic factor characteristics and contribute to intergenerational inheritance of high fat diet-induced metabolic disorders, and the tRNA methyltransferase, DNA methyltransferase 2, may be involved in this process [34,68,69]. Lastly, in female animals, tRNA fragments are present at diverse levels between preeclamptic and normal trophoblast debris. These small RNAs may be transferred into endothelial cells and then impact preeclampsia via feto-maternal signaling [70].

### 4.4. Regulating Immunity and Disease

As described above, tRFs mediate viral immune evasion from the host immune system. Additionally, tRNA fragments may regulate immunity considering their role in repressing gene expression and the abundance of tRFs and tiRNAs in immune cells. Indeed, Wang et al. identified a tRNA-Ala (UGC) fragment that mediates the T helper 1 immune response, and is also necessary for reorganization by toll-like receptor 3 (TLR3) in BALB/c mice infected with hepatitis B [71]. According to sequencing results, tRF-3 is the most abundant subset of tRNA fragments in mature B cells in lymphoma cell lines. CU1276, a differentially expressed tRF-3 fragment, is downregulated and its low abundance represses endogenous replication protein A1 (PRA1) and promotes cell growth and proliferation [72]. td-piR (Glu) downregulates CD1a expression as the promoter region is modified by H3K9me3 methylation and this reaction is attenuated by interleukin-4 during lipid antigen presentation in dendritic cells [73]. Extracellular vesicles (EVs) are a robust source of T cell activation, and their activation also selectively releases selective tRFs into EVs, indicating a novel intercellular communication mode for repressing immune activation [74]. tRF-3 and 5′-tRNA halves are both abundant and highly dynamic in placental/decidual tissue over time to counter maternal immune activation in a maternal-immune-activation autism model in mice [75]. 5′-tRFs are immune signaling molecules that support cell-to-cell communication in hematopoietic and lymphoid cells in humans and mice [76]. Lastly, tRFs and tiRNAs are involved in the mucosal immune response by regulating different pathways [77].

tRNA-derived fragments are essential in metabolic diseases, cancers, and neurodegenerative diseases (Table 1). Despite its diverse roles in cardiac hypertrophy, as described above, tRF-3001b is also highly expressed in non-alcoholic fatty livers in mice and tRF-3001b overexpression inhibits the autophagy-related gene Prkaa1 and impacts autophagy, ultimately promoting development of the disease [78]. Identifying biomarkers for the early diagnosis of cancer has proven difficult. However, recent high-throughput sequencing has revealed several tRNA fragments that are widely observed in cancers as novel biomarkers. For example, the tRNA-derived fragment tRF-21 is a potential suppressor of pancreatic ductal adenocarcinoma via mRNA alternative splicing both in vivo and in vitro, which may be clinically applied [78]. tRF-5026a is involved in the PTEN/PI3K/AKT signaling pathway to inhibit the proliferation of gastric cancer cells [79]. Differentially expressed tRFs also regulate cell invasion, migration, and proliferation in breast, gastric, ovarian and other cancers, and specific tRFs show high correlation with receiver operating characteristic curves [14,24,30,80,81,82,83]. 5′-tRNA-4-Val-AAC, a tRNA half fragment, also demonstrates a significant positive correlation with clear cell renal cell carcinoma [84].

Considering their important regulatory functions, several databases, including tRF2cancer and OncotRF, have been established to better understand the molecular mechanisms of tRFs, in addition, The Cancer Genome Atlas (TCGA) contains bioinformatics of tRFs sequencing results of several cancers [85,86,87]. ANG-mediated tRNA fragments are widely detected and associated with neurodegenerative diseases [88]. Hogg et al. [89] found that tRF-5 expression from Gly-GCC increases in epilepsy and decreases after neuronal hyperexcitability. Specific tRFs have also been identified in both patients with Parkinson’s disease and healthy controls at distinct abundances, which may serve as sex-dependent biomarkers, particularly to distinguish between Parkinson’s disease with or without dementia [90]. A similar difficulty in Alzheimer’s disease (AD) diagnosis is the lack of biomarkers because the AD mechanism remains unclear. tRF5-Cys-GCA and tRF5-Pro-AGG correlate with AD, and tRF expression in AD is upregulated in response to an NOP2/Sun RNA methyltransferase 2 (NSUN2)-mediated decrease in tRNA methylation [91]. Cleavage factor polyribonucleotide kinase subunit 1 (CLP1) mutation links cerebellar development and functions in tRNA splicing and cell metabolism. 5′-tRF-Tyr accumulates in the brains of newborn mice in pontocerebellar hypoplasia type 10 caused by a CLP1 R140H mutation, indicating that increases in 5′-tRF-Tyr occur during the developmental phase [92] (Table 1). A CAG trinucleotide repeat mutation in the huntingtin gene results in Huntington disease (HD), with similar pathology in mice and humans caused by the derived small RNAs with the CAG repeat. The inhibition of CAG specifically mitigates HD-sRNA-PT neurotoxicity, indicating that these alanine-derived tRFs may be used in treating HD [93]. Aging-induced cellular stress is a risk factor for neurodegenerative disease. 5′- and 3′-tRFs display dynamic changes with age in rat brains, such that 3′-tRFs range in a narrow size and consistently increase from 6 to 22 months of age, while 5′-tRFs decrease from 6 to 12 months and then rise at 22 months [94]. The distribution of 5′-tRNA halves is also tissue-specific, in which 5′ tRNA halves are primarily generated in hematopoietic-related diseases and changes with age are mitigated by calorie restriction [95]. These data support further exploration of the roles of tRFs in neurodegeneration. 

tRFs are present in the cytoplasm as key messengers that connect nuclear and mitochondrial communication [96]. 

Mitochondria supply ATP via the oxidative phosphorylation (OXPHOS) system in eukaryotic cells. It has been proved that mitochondrial dysfunction activates retrograde signaling pathways from the mitochondria to the nucleus which results in OXPHOS disease. MELAS (mitochondrial encephalomyopathy, lactic acidosis, and stroke-like episodes), which is a typical OXPHOS disease, was mostly caused by mutation m.3243A>G in the mitochondrial tRNALeu(UUR) gene. Considering some evidence showed that deregulation of small non-coding RNAs by pathological mtDNA mutation were responsible for nuclear gene expression [97], tRFs may be involved in this as well. Based on sequencing results, it was found that m.3243A>G significantly altered the expression of mt tRFs, especially mt itRF GluUUC, which acted as a post-transcriptional regulator of a nuclear gene (MPC1) [98]. Exosomes could act as extracellular vesicles (EVs) to regulate cell-to-cell communication by releasing small non-coding RNAs. Recent research proved that tRF-25, tRF-38, and tRF-18 in plasma exosomes demonstrated better accuracy for the diagnosis of gastric carcinoma [99]. tRFs in EVs are also important for cell-to-cell communication [100]. tRFs in EVs perform similar biological functions using similar molecular mechanisms as intercellular tRFs. tRF-22-8BWS7K092 derived from exosomes promotes ferroptosis in alveolar epithelial cells by activating the Hippo signaling pathway in acute lung injury [101]. However, compared with that of intercellular tRFs, the generation mechanism and the methodological isolation details of tRFs in EVs require further exploration. In summary, tRNA fragments exhibit close relationships with the onset and progression of metabolic diseases, cancers, and neurodegenerative diseases. Thus, they may be novel therapy targets following their deliberate evaluation.

**Table 1:** Different types of tRFs in diseases [102].

tRFs ID/Name	Type	Disease/Cell Model	Cell Function	Reference
tRF-1001	tRF-1	Prostate cancer	Promotes cell proliferation	[23]
ts46	tRF-1	Breast cancer	Upregulated with the mutation of PIK3CA	[13]
tRF^GluYTC^	i-tRF	Breast cancer	Destabilization of YBX-1 bound oncogenic transcripts suppress cell proliferation and cancer metastasis	[29]
tRF^Val^	tRF-5	Liver cancer	Cleaves tRNAs during stress	[64]
tRF^HisGTG^	tRF-3	B cell lymphoma	Associates with Ago2 and downregulates target genes by transcript cleavage	[25]
tRF-5026a	/	Gastric cancer	Inhibits proliferation	[79]
tRF^GlyGCC^	tRF-5	epilepsy	Biomarker	[89]
tRF5-Pro-AGG	tRF-5	Alzheimer’s Disease:	Age- and stage-dependent	[91]
5′-tRF-Tyr	tRF-5	Pontocerebellar hypoplasia type 10	Involved in cleavage factor polyribonucleotide kinase subunit 1 (CLP1) mutation	[92]

### 4.5. tRNA Fragments in Oxidative Stress and Apoptosis

tRNA fragmentation is a conserved modification type in biological processes. Increasing evidence reveals that tRNA fragments are important regulators that help cells survive against stress. tRNA cleavage was first identified in *C. elegans* and yeast in response to oxidative stress. In *S. cerevisiae*, the RNase Rny1p relocalizes to resist oxidative stress, allowing for tRNA cleavage and promoting cell death. This circuit resembles the mitochondrial approach to induce apoptosis followed by cytochrome c release [20]. As tRNA cleavage is conserved among eukaryotes, oxidative stress-induced tRNA-Tyr-GUA fragment generation has been demonstrated in human mammary epithelial cells. Moreover, depletion of these tRNA-Tyr-GUA fragments represses cell proliferation by inhibiting heterogeneous nuclear ribonucleoprotein A1 stability in a DIS3L2-dependent manner [103]. Oxidative stress triggers tRNA cleavage in Arabidopsis, which is also observed in HeLa and ARPE-19 human cell lines [22]. These findings suggest that tRNA fragments modulate cell fate in pathological processes. For instance, surgery is one of the most common treatments for neurological and metabolic diseases; however, ischemic-reperfusion injury may simultaneously occur with cellular oxidative stress, which is harmful to the body. Component 3 activation-mediated Gly-tRFs interact with AGO-3 and downregulate sirtuin 1 expression in alcoholic fatty liver disease [104]. tRNAs undergo numerous chemical modifications to monitor transcription and translation. The cytosine-5 RNA methyltransferase NSUN2 is repressed by oxidative stress, while loss of NSUN2 determines the biogenesis of tRFs [105].

The 5′-tiRNA contains a 2′,3′-cyclic phosphate (cP), and this RNA cleavage is highly specific. Oxidative stress results in the accumulation of these 2′,3′-cP-containing RNAs [106]. 5′-tiRNAs promote stress-induced SG assembly, and 5′-monophosphate is strictly required for this process [19]. These findings suggest that tRNA fragments may rely on biochemical structure and conformation in the cellular stress response. High levels of metabolism are required to maintain the proliferation and differentiation of T cells, whereas a high level of reactive oxygen species (ROS) is produced paradoxically. Yue et al. [107] determined that Schlafen 2 deficiency in T cells increases ROS levels and triggers oxidative stress, followed by tRNA fragment accumulation and inhibition of translation and promotion of SG formation. Similar results have been observed in oxidative stress damage in rat neuronal PC12 cells, in which tiRNAs were detected in neuronal cells under stress conditions, and tiRNA generation was closely associated with cell damage and death. Moreover, the functions of CLP1 are complicated in vivo, and CLP1 knockout mice exhibit multiple neuronal loss behaviors. Lastly, the activity loss of CLP1 results in Tyr-tRNA fragment accumulation, which is more sensitive to oxidative stress and H_2_O_2_-induced cell death via a p53-dependent pathway [108].

In addition, tRNA fragments impact apoptosis. The 3′-tRNA-derived fragment, tRF-Val, inhibits apoptosis by binding to the chaperone molecule, eukaryotic translation elongation factor 1 alpha 1, directly and inhibiting the downstream p53 molecular pathway in gastric cancer [109]. Furthermore, tRF-315 derived from tRNA-Lys alleviates cisplatin-induced mitochondrial dysfunction and apoptosis in prostate cancer [110]. Other tRFs rescue cell apoptosis by binding to cytochrome c when it is under sodium arsenite stress in Hela cells [111]. tiRNAs may also be responsible for apoptosis. Inhibiting tiRNA-Gly-CCC/GCC leads to mitochondrial dysfunction and enhances palmitic acid-induced apoptosis in human trophoblasts [112]. These findings provided new insights that tRFs and tiRNAs were involved in apoptosis induced by diverse stress conditions.

## 5. Conclusions and Perspectives

Taken together, tRFs and tRNA halves are new hotspots in non-coding RNA research. Increasing identification of tRFs and tRNAs in various biological processes and molecular mechanisms in transcription, translation, epigenetic regulation, and other physiological activities improves our understanding of these molecules. However, research on tRFs and tRNA halves is still preliminary; in-depth investigation is required to clarify their regulatory functions.

Currently, the biogenesis mechanism of tRFs and tRNA halves is controversial. However, RNase Z, Dicer, and ANG are confirmed factors in tRF and tRNA half generation; yet, the ribonuclease responsible for tRF-2 generation remains unclear. Furthermore, controversy surrounds whether tRF-3s are generated from stem–loop hairpin secondary structures or standard cloverleaf tRNA forms, which suggests that tRNA modifications may influence the biogenesis of tRFs and tRNA halves.

Due to the development of high-throughput sequencing technologies, novel tRFs and tRNA halves have been identified in different physiological and pathophysiological environments; however, a systematic naming convention is lacking. The current databases focus on humans, particularly on cancers. In contrast, the abundance and composition of tRFs and tRNA halves is dependent on species, stress types, and subcellular localization. Therefore, databases that include other physiological or pathological models and species are urgently needed.

In summary, studies on tRFs and tRNA halves enable a broader understanding of this novel class of small non-coding RNA regulation. tRFs and tRNA halves shown promising potential targeting functions in physiological and pathological conditions and provide novel insight into the molecular mechanism of life.
